# Ribosomal Protein S6 Kinase (RSK)-2 as a central effector molecule in RON receptor tyrosine kinase mediated epithelial to mesenchymal transition induced by macrophage-stimulating protein

**DOI:** 10.1186/1476-4598-10-66

**Published:** 2011-05-28

**Authors:** Qi Ma, Sunny Guin, Snehal S Padhye, Yong-Qing Zhou, Rui-Wen Zhang, Ming-Hai Wang

**Affiliations:** 1 Division of Cancer Biology at State Key Laboratory for Diagnosis & Treatment of Infectious Diseases, First Affiliated Hospital, Zhejiang University College of Medicine, Hangzhou, 310003, China; 2 Cancer Biology Research Center and Department of Biomedical Sciences, School of Pharmacy, Texas Tech University Health Sciences Center, Amarillo, TX 79106, USA; 3 Department of Neurosurgery, First Affiliated Hospital, Zhejiang University College of Medicine, Hangzhou, 310003, China; 4 Department of Pharmaceutical Sciences, School of Pharmacy, Texas Tech University Health Sciences Center, Amarillo, TX 79106, USA

## Abstract

**Background:**

Epithelial to mesenchymal transition (EMT) occurs during cancer cell invasion and malignant metastasis. Features of EMT include spindle-like cell morphology, loss of epithelial cellular markers and gain of mesenchymal phenotype. Activation of the RON receptor tyrosine kinase by macrophage-stimulating protein (MSP) has been implicated in cellular EMT program; however, the major signaling determinant(s) responsible for MSP-induced EMT is unknown.

**Results:**

The study presented here demonstrates that RSK2, a downstream signaling protein of the Ras-Erk1/2 pathway, is the principal molecule that links MSP-activated RON signaling to complete EMT. Using MDCK cells expressing RON as a model, a spindle-shape based screen was conducted, which identifies RSK2 among various intracellular proteins as a potential signaling molecule responsible for MSP-induced EMT. MSP stimulation dissociated RSK2 with Erk1/2 and promoted RSK2 nuclear translocation. MSP strongly induced RSK2 phosphorylation in a dose-dependent manner. These effects relied on RON and Erk1/2 phosphorylation, which is significantly potentiated by transforming growth factor (TGF)-β1, an EMT-inducing cytokine. Specific RSK inhibitor SL0101 completely prevented MSP-induced RSK phosphorylation, which results in inhibition of MSP-induced spindle-like morphology and suppression of cell migration associated with EMT. In HT-29 cancer cells that barely express RSK2, forced RSK2 expression results in EMT-like phenotype upon MSP stimulation. Moreover, specific siRNA-mediated silencing of RSK2 but not RSK1 in L3.6pl pancreatic cancer cells significantly inhibited MSP-induced EMT-like phenotype and cell migration.

**Conclusions:**

MSP-induced RSK2 activation is a critical determinant linking RON signaling to cellular EMT program. Inhibition of RSK2 activity may provide a therapeutic opportunity for blocking RON-mediated cancer cell migration and subsequent invasion.

## Background

Epithelial to mesenchymal transition (EMT) is a biological process in polarized epithelial cells, which occurs in various physiological and pathological conditions [1]. Complete EMT is characterized by spindle-like cell morphology, loss of epithelial cellular markers such as E-cadherin, and gain of mesenchymal phenotype by expressing filament proteins including vimentin and α-smooth muscle actin [1,2]. Cells undergoing EMT are highly mobile and invasive [2,3]. During embryonic development, EMT enables cells to migrate or invade into neighboring tissues and maturate or differentiate into specialized cells [1,2]. In epithelial malignant progression, EMT has emerged as a critical player in regulating cancer cell invasive phenotype [4,5]. Acquiring EMT is a critical step for cancer cells to dissociate from a primary tumor mass and subsequently migrate and invade adjacent tissues for remote metastasis [4,5]. Recently, EMT has been linked with cancer stem-like phenotype in certain epithelia tumors [6,7]. As demonstrated, breast cancer cells express several cellular markers that resemble the stem-like phenotype during their progression towards EMT [6,7]. These observations highlight the importance of cellular EMT program in tumorigenic progression of cancer cells.

Development of EMT in cancer cells is regulated and precisely controlled at different cellular levels [4,5]. Various proteins such as receptor tyrosine kinases (RTK) [8-10], cytokine receptors [11,12], intracellular signaling molecules [13,14], and transcriptional factors [15,16] are involved in cellular EMT program. At the signaling level, RTK-mediated activation of extracellular signal-regulated kinase (Erk1/2) has been implicated as a critical pathway for initiation of EMT [13,17,18]. Transforming growth factor (TGF)-β1-stimulated TGF-β receptor I/II and Smad signaling also play a pivotal role in induction of EMT [11,19]. Additional pathways such as Wnt-β-catenin signaling also have been implicated in EMT [20]. Convincing evidence indicates that signals coordinated among different pathways such as the RTK-Erk1/2 and TGF-β1-Smad pathways maximize trans-differentiation of epithelial tumor cells towards EMT [1,2]. Moreover, such coordination raises the possibility that a converging signal for diverse pathways may exist, and may act as a central determinant controlling cellular EMT program.

Human 90 kDa ribosomal S6 kinases (RSK) belong to a family of Ser/Thr kinases with two unique functional kinase domains [21]. The family consists of four isoforms (RSK1-4), of which RSK1 and RSK2 are currently under intensive investigation for their roles in cellular signaling [21-23]. In quiescent cells, RSK forms a protein-protein complex with Erk1/2 [24] and is considered to be a downstream signaling molecule of the Ras-Erk1/2 pathway [21]. Activation of RSK is featured by phosphorylation, dissociation from Erk1/2, and subsequent nuclear translocation [21]. Various extracellular factors including growth factors, cytokines, chemokines, peptide hormones, and neurotransmitters are known to directly activate RSK [21]. RSK phosphorylation occurs at multiple Ser and Thr residues through sequential steps by various kinases such as Erk1/2 [21-24]. Activated RSK phosphorylates many cytosolic and nuclear targets such as FLNA, BAD, DAPK, p27^KIP1^, and transcription factors including CREB, NF-κB, and NFAT3 [21-25]. Recently, RSK has emerged as a major player in the control of epithelial cell phenotype and motility [22]. RSK is indicated as a principal effector of the Ras-Erk1/2 pathway for eliciting a coordinated promotile/invasive program and phenotype in epithelial cells [22]. A genome-wide RNAi screen also has found that multiple proteins in various pathways depend on RSK for cellular migration [23]. These discoveries indicate that activation of RSK could be an essential convergent point for regulating cellular phenotypic changes and motile/invasive activities.

The present study sought to identify the major signaling molecule(s) responsible for EMT induced by macrophage-stimulating protein (MSP) [26], also known as hepatocyte growth factor (HGF)-like protein [27]. MSP is a serum-derived growth factor that specifically binds and activates the RON receptor tyrosine kinase [28,29], a member of the MET proto-oncogene family [27]. Previous studies have observed that RON-mediated activation of the Ras-Erk1/2 pathway is critically important in transducing signals leading to EMT [30,31]. However, the downstream signaling molecule(s) that controls RON-mediated EMT is unknown. To facilitate this study, Martin-Darby canine kidney (MDCK) cells expressing human RON, which is known to show complete EMT [30,31] was used as a model and a cell-shape based screen using various small chemical inhibitors was applied. By analyzing potential signaling proteins that are involved in MSP-induced EMT-like activities, we discovered that RSK2 is a principle effector molecule responsible for MSP-induced EMT in MDCK and human cancer cells. Evidence also indicates that RSK2 is responsible for TGF-β1-induced EMT.

## Materials and methods

### Cell Lines and Reagents

Martin-Darby canine kidney (MDCK) and human colon cancer HT-29 cells were purchased from ATCC (Manassas, VA). MDCK cells stably expressing RON (M-RON) were established as previously described [30]. Human pancreatic cancer L3.6pl cells were provided by Dr. G. E. Gallick (University of Texas M.D. Anderson Cancer Center, Houston, TX). Human MSP was provided by Dr. E. J. Leonard (National Cancer Institute, Bethesda, MD). Human transforming growth factor (TGF)-β1 was from R&D (Minneapolis, MN). Mouse monoclonal antibodies (mAb, clone Zt/g4) and rabbit IgG antibody (R5029) were used as previously described [32]. Mouse mAb specific to phospho-tyrosine (PY-100), phospho-Erk1/2, and other signaling proteins were from Cell Signaling (Danvers, MA). Mouse, rabbit, or goat IgG antibodies specific to panRSK, RSK1, RSK2, Snail, E-cadherin, vimentin, claudin-1, and F-actin were from BD Transduction Laboratories (Lexington, KY). PD98059 (PD), wortmannin (WT), U0126, SB203580, SB431524, rapamycin, and SL0101 were from CalBiochem (San Diego, CA). Small molecule inhibitor Compound-1 (CP-1) specific to human RON [33] was from Amgen (Thousand Oak, CA). SP600125, S31-201, XAV-939, vismodegib, and SB431542 were from Selleck Chemicals (Houston, TX), and Cay10512 was from Cayman Chemicals (Ann Arbor, MI).

### Transient expression of human RSK1 or RSK2 in HT-29 cells

Transfection of cells with pcDNA3.1 containing RSK1 (pRSK1) or RSK2 cDNA (pRSK2) was performed using Lipofectamine as previously described [32]. Briefly, cells (1 × 10^6 ^cells in 60 mm diameter dish) were cultured overnight and then transfected with 3 μg/dish of pRSK1 or pRSK2 vectors. The pRSK1/2 plasmids were provided by Dr. J. Chen (Emory University School of Medicine, Atlanta, GA). Cells transfected with an empty vector pcDNA3.1 were used as control. Transfected cells were incubated for 48 h and then processed for various biological assays.

### Immunoprecipitation and Western blot analysis

These methods were performed as previously described [30]. Cellular proteins (250 μg/sample) were used for immunoprecipitation by Zt/g4 (1 μg/sample) coupled to protein G Sepharose beads. Individual proteins were detected using specific antibodies in Western blot analysis under reducing conditions. Membranes were reprobed with rabbit IgG antibody to β-actin to ensure equal sample loading [30].

### Cellular immunofluorescent analysis

The method was performed as previously described [30]. To detect cytoplasmic or nuclear proteins, cells at 1 × 10^4 ^cells per well in a 24-well plate were cultured overnight and then stimulated for 24 h with MSP, TGF-β1 or both in the presence or absence of various small chemical inhibitors. Cells were fixed with cold acetone and incubated with specific antibodies, followed by goat anti-mouse IgG coupled with FITC. Normal mouse IgG was used as the negative control. Cellular immunofluorescence was observed under Olympus BK71 microscope equipped with fluorescent apparatus as previously described [30].

### Methods for silencing RSK1 or RSK2 mRNA expression in L3.6pl cells

Synthetic siRNA specific to human RSK1 or RSK2 were acquired from Dhamacon (Chicago, IL). To knockdown RSK expression, L3.6pl cells were cultured overnight and then transfected with RSK1 or RSK2 siRNA according to the manufacturer's instructions. After incubation for 48 h, cells were washed and then processed for biochemical and biological analyses.

### Assays for cell morphological changes

The assays were performed as previously described [30]. M-RON or other cells (2 × 10^4^/well in a 24-well plate) were cultured overnight and then stimulated with or without MSP (2 nM), TGF-β1 (5 ng/ml), or both at 37°C for 24 h. Cell morphological changes were observed and photographed using an Olympus BK71 inverted microscope equipped with CCD camera. The length of individual cells from experimental groups was determined by measuring 200 cells and results were expressed as elongation index and compared among various groups [30].

### Cell migration assays

Wound healing assay was used to determine the ability of cells to migrate and fill the open space as previously described [32]. Cells were stimulated with MSP (2 nM), TGF-β1 (5 ng/ml) or both for 16 or 24 h. The percentage of open space filled by migrated cells was calculated as previously described [32].

## Results

### Identification of RSK as an effector molecule in RON-mediated EMT using cell-shape change based screen by various small chemical inhibitors

MSP induces complete EMT in MDCK cells, featured by spindle-like morphology, diminished E-cadherin expression, appearance of mesenchymal marker vimentin, and increased cell migration and invasiveness [30,31]. However, the major signaling molecule(s) linking RON signaling to these changes is unknown. To identify these molecules, we performed a MSP-induced cell-shape based screen using a panel of 12 small chemical inhibitors in M-RON cells. Intracellular proteins representing 10 signaling pathways such as Erk1/2, PI-3 kinase, β-catenin, Stat3, NF-κB and others were targeted. These signaling proteins are known to be involved in cell morphological changes and motility [30,34-37]. Cell elongation index measured from spindle-like morphology was used to determine the effect of individual inhibitors (Table 1). Prevention of MSP-induced spindle-like morphology was not observed in M-RON cells treated with wortmannin, SB203580, SP600125, Cay10512, and S31-201, suggesting that signaling from these pathways was not involved in MSP-induced EMT. A moderate effect, based on changes in elongation index, was seen when rapamycin, vismodegib, and XAV-939 were applied, suggesting that signaling from Hedgehog, Wnt/β-catenin, and FRAP/mTOR pathways played a role in MSP-induced EMT. As expected, inhibition of RON and Erk1/2 signals by CP-1 and PD98059, respectively, completely blocked the effect of MSP, indicating the importance of the RON-Erk1/2 pathway in regulating EMT phenotype. An interesting result was the outcome of SL0101-mediated effects, which completely prevented MSP-induced EMT. SL0101 is a specific inhibitor of RSK and regulates various cellular activities [38]. The observed effects prompted us to determine if RSK is indeed a critical determinant in RON-mediated EMT.

**Table 1:** Effect of Various Small Chemical Inhibitors on MSP-Induced Spindle-Like Morphologies in RON-Expressing MDCK cells*

Small molecule inhibitors	Signal proteins inhibited	Amount used to treat cells	Cell elongation index	Preventive effect (%)
**Control**	N/A	N/A	1.0 ± 0.24	None
**MSP**	none	2.0 nM	3.12 ± 0.47	N.D
**CP-1**	RON	100 μM	1.05 ± 0.26	**98.4%**
**PD98059**	MEK1/2	100 μM	1.03 ± 0.31	**97.1%**
**Wortmannin**	PI-3 kinase	50 μM	2.9 ± 0.41	7.1%
**SB203580**	p38 MAP kinase	50 μM	3.1 ± 4.6	0.6%
**SP600125**	JNK1,2,3	5 μM	3.07 ± 0.39	1.6%
**Cay10512**	NF-κB	5 μM	2.03 ± 0.33	39.4%
**S31-201**	Stat3	10 μM	1.25 ± 0.34	59.9%
**XAV-939**	Wnt/β-catenin	5 μM	2.16 ± 0.32	30.8%
**SL0101**	RSK	50 μM	1.09 ± 0.27	**97.1%**
**Rapamycin**	FRAP/mTOR	100 nM	2.69 ± 0.25	13.8%
**Vismodegib**	Hedgehog	1 μM	2.04 ± 0.22	34.6%
**SB431542**	TGF-β1 receptor	1 μM	2.97 ± 0.24	4.8%

*M-RON cells (1 × 10^4 ^cells/well) in DMEM containing 1% FBS were stimulated with 2 nM of MSP in the presence of absence of individual small chemical inhibitors for 24 h. Cell morphological changes were observed under microscope and photographed with CCD camera. Cell elongation index (CEI) was determined by measuring the length of individual adherent cells among various groups. CEI from control cells was set as one. The percentages of preventive effect were calculated by comparing with as the CEI from MSP-treated cells previously described [45]

### MSP-induced RSK2 dissociation with Erk1/2 and its phosphorylation in correlation with Erk1/2 activation

**Figure 1:**
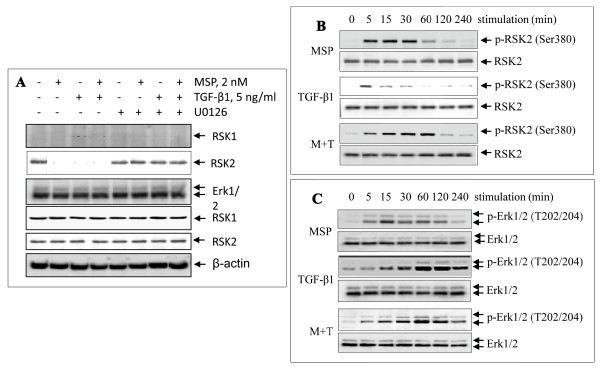
**MSP-induced RSK2 dissociation with Erk1/2 and their phosphorylation in M-RON cells**. **A**) MSP-induced dissociation of RSK2 from Erk1/2 in intact cells: M-RON cells (3 × 10^6 ^cells/dish) were incubated in DMEM containing 1% FBS overnight and then stimulated for 30 min with MSP (2 nM), TGF-β1 (5 ng/ml), or both in the presence or absence of 5 μM of U0126. Cellular proteins (250 μg/sample) from cell lysates were subjected to immunoprecipitation with rabbit IgG antibody specific to Erk1/2. Proteins in anti-Erk1/2 immunocomplex were subjected to Western blot analysis using antibodies specific to RSK1 or RSK2. Membranes were also reprobed with IgG antibody to Erk1/2 as the loading control. **B**) and **C**) MSP-induced RSK2 phosphorylation and its correlation with Erk1/2 activation: M-RON cells (3 × 10^6 ^cells/dish) in DMEM with 1% FBS were stimulated with MSP, TGF-β1, or both for various times. Cellular proteins (50 μg/sample) from cell lysates were subjected to Western blot analysis. Phosphorylation of RSK2 and Erk1/2 was detected by individual antibodies specific to phospho-RSK2 Ser380 or Erk1/2 T202/204, respectively. RSK2 and Erk1/2 detected by their corresponding regular antibodies were used as the loading control.

RSK isoforms such as RSK1 or RSK2 associate with Erk1/2 in quiescent cells [21]. Dissociation between RSK and Erk1/2 requires phosphorylation [21]. To determine which RSK isoform(s) is regulated by MSP, M-RON cells were stimulated in the presence or absence of U0126, an inhibitor that blocks RSK dissociation with Erk1/2 [39]. TGF-β1 was used as the control. RSK isoforms associated with Erk1/2 were determined by anti-Erk1/2 mAb immunoprecipitation followed by Western blot analysis using anti-RSK1 or RSK2 antibody. As shown in Figure 1A, RSK2 but not RSK1 was spontaneously associated with Erk1/2 in M-RON cells cultured in DMEM containing 1% FBS. In contrast, interaction between RSK1 and Erk1/2 was not observed. It should be pointed out that RSK1 was expressed in M-RON cells (data not shown); however, Erk1/2 was not detected in anti-RSK1 immunoprecipitation. After MSP stimulation, RSK2-Erk1/2 complex dissociated. TGF-1β also induced RSK2-Erk1/2 dissociation although its effect was moderate. However, in cells treated with U0126, MSP or MSP plus TGF-β1-induced dissociation of RSK2-Erk1/2 complex was blocked. Similar results were observed when immunoprecipitation was performed using anti-RSK2 mAb (data not shown). Taken together, these results suggested that MSP is capable of regulating RSK2 interaction with Erk1/2 and TGF-β1 exerts a similar effect. MSP-induced dissociation could be the first step in regulating RSK2 activity.

The next experiment determined whether MSP activates RSK2 in association with Erk1/2 phosphorylation. Again, TGF-β1 was used for comparison. Results in Figure 1B showed the time-dependent RSK2 phosphorylation at Ser380 residue. MSP acted as a strong inducer of RSK2 phosphorylation, in which high levels of RSK2 phosphorylation were maintained for up to 30 min and then gradually reduced. The effect of TGF-β1 on RSK2 phosphorylation was relatively weak, which peaked at about 5 min and then gradually diminished. In combined stimulation, TGF-β1 significantly potentiated MSP-induced RSK2 phosphorylation. In this case, RSK2 phosphorylation was prolonged up to 60 min, a significant increase compared to those stimulated by MSP or TGF-β1alone.

To correlate RSK2 phosphorylation with Erk1/2 activation, we determined MSP or TGF-β1-induced Erk1/2 phosphorylation. Results in Figure 1C showed that MSP strongly induced Erk1/2 phosphorylation at Tyr 202/204 residues. Significant Erk1/2 phosphorylation was seen as early as 5 min, peaked at 15 min, and then gradually reduced to the baseline at 240 min (4 h). Such a time-dependent kinetic effect correlated well with the time course of RSK2 phosphorylation (Figure 1B). In contrast, TGF-β1-induced Erk1/2 phosphorylation occurred at relatively later stages and had a delayed time course. The curve did not seem to correlate with the time course of RSK2 phosphorylation (Figure 1B). Again, TGF-β1 potentiated MSP-induced Erk1/2 phosphorylation. A strong and long-lasting effect on Erk1/2 phosphorylation was achieved when both stimuli were used (Figure 1C). These results, together with those shown in Figure 1B, demonstrated that MSP is a strong inducer of RSK2 phosphorylation. The kinetics of phosphorylation between Erk1/2 and RSK2 correlated well upon MSP stimulation. TGF-β1 showed a moderate stimulating effect on RSK2 phosphorylation. It induced Erk1/2 phosphorylation but showed a relatively delayed time-course. However, TGF-β1 potentiated MSP-induced RSK2 and Erk1/2 phosphorylation.

### Prevention of MSP-induced RSK2 activation by small chemical inhibitors specific to RON and Erk1/2

**Figure 2:**
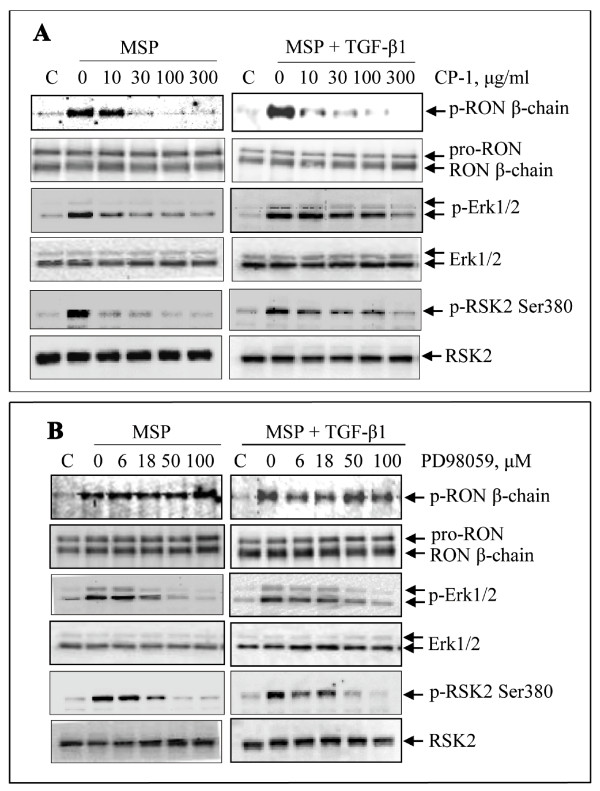
**Inhibitory effect of CP-1 and PD98059 on MSP or MSP plus TGF-β1-induced RSK2 phosphorylation**: M-RON cells (3 × 10^6 ^cells per dish) in DMEM with 1% of FBS were first treated with CP-1 or PD98059 for 10 min followed by stimulation with MSP (2 nM) or MSP plus TGF-β1 (5 ng/ml). Cells were collected 30 min after stimulation. Phosphorylated RON was determined by Western blot analysis after Zt/g4 immunoprecipitation of cell lysates (250 μg proteins/sample). Phosphorylated Erk1/2 and RSK2 were directly determined by Western blot analysis using specific antibodies, respectively. Membranes were also reprobed with individual antibodies to detect non-phosphorylated proteins as the loading controls. Data shown here are from one of two experiments with similar results.

To determine if MSP-induced RSK2 phosphorylation is indeed mediated by RON and Erk1/2 signaling, M-RON cells were stimulated in the presence or absence of specific RON inhibitor CP-1 and Erk1/2 inhibitor PD98059. RSK2 phosphorylation was determined by Western blot analysis. CP-1 inhibited MSP-induced RON phosphorylation in a dose-dependent manner (Figure 2A). CP-1 treatment also led to diminished Erk1/2 phosphorylation. Significantly, CP-1 inhibited MSP-induced RSK2 phosphorylation in a dose-dependent manner. We also observed the inhibitory effect of CP-1 in cells stimulated with MSP plus TGF-β1. However, levels of inhibition, as shown by the phosphorylation levels of Erk1/2 and RSK2, were not as strong as those shown in cells stimulated with MSP alone. Dramatic inhibition was only seen when high concentrations of CP-1 (up to 300 μg/ml) were used. Results from PD98059 experiments confirmed that inhibition of Erk1/2 had no effect on MSP-induced RON phosphorylation. However, levels of Erk1/2 phosphorylation were diminished by PD98059 in a dose-dependent manner (Figure 2B). Moreover, PD98059 inhibited MSP or MSP plus TGF-β1-induced RSK2 phosphorylation in a dose-dependent manner. Thus, the results in Figure 2 demonstrated that by inhibiting RON or Erk1/2 activation, both CP-1 and PD98059 are able to prevent MSP or MSP plus TGF-β1-induced RSK2 phosphorylation, suggesting that activated RON and Erk1/2 signaling is required for MSP-induced RSK2 phosphorylation.

### Effect of MSP on RSK2 nuclear translocation and phosphorylation

**Figure 3:**
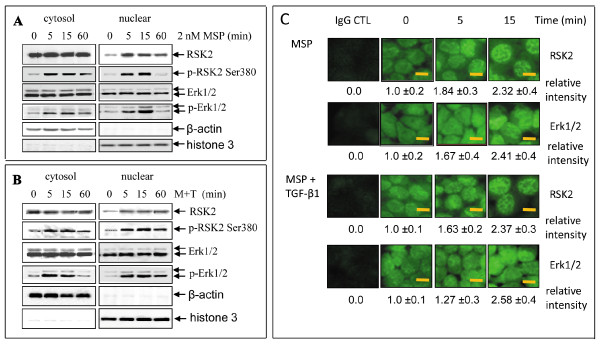
**Effect of MSP on RSK2 phosphorylation and its nuclear translocation**: M-RON cells (3 × 10^6 ^cells per dish) in DMEM with 1% FBS were stimulated with MSP (2 nM), TGF-β1 (5 ng/ml), or MSP plus TGF-β1 for various times. Cellular proteins (50 μg/sample) from cell lysates were subjected to Western blot analysis using antibodies specific to regular or phospho-RSK (Ser380) and Erk1/2. **A**) MSP induced RSK2 phosphorylation and nuclear translocation. **B**) Effect of MSP and TGF-β1 on RSK2 phosphorylation and nuclear translocation. **C**) Immunofluorescent analysis of MSP or MSP plus TGF-β1-induced RSK2 nuclear translocation. M-RON cells were stimulated with MSP or MSP plus TGF-β1v for various times. Cells were fixed with cold acetone, blocked with 1% BSA, followed by incubation with antibody specific to RSK2. FITC coupled rabbit anti-mouse IgG was used as the detecting antibody. Immunofluorescence was observed by Olympus BT71 microscope equipped with fluorescent apparatus as previously described [45]. Scale bars represent 5 μm.

To further determine the effect of MSP on RSK2, we studied RSK2 nuclear translocation in comparison with Erk1/2 activation. Cells were stimulated by MSP or MSP plus TGF-β1 for various times and cytoplasmic and nuclear proteins were prepared. RSK2 was mainly detected in cytoplasmic fraction in non-stimulated M-RON cells. A small amount of RSK2 was also present in nuclear proteins (Figure 3A). This pattern was similar to that of Erk1/2, in which Erk1/2 in both cytoplasmic and nuclear fractions was observed. Upon MSP stimulation, the amounts of RSK in nuclear fraction were dramatically increased in a time-dependent manner. Phosphorylation was observed not only in cytosolic but also in nuclear RSK2. Again, a similar pattern was documented for Erk1/2, in which phosphorylated Erk1/2 was detected in nuclear proteins. Results in Figure 3B demonstrated that MSP in combination with TGF-β1 induced RSK2 nuclear translocation and phosphorylation. This effect was accompanied by Erk1/2 phosphorylation. A major difference was that the time course for both RSK2 and Erk1/2 phosphorylation lasted longer in MSP and TGF-β1 co-stimulated cells than in cell treated with MSP alone.

We further validated results from Western blotting by studying cellular RSK and Erk1/2 distribution using DSU confocal microscope image analysis. Cytoplasmic and nuclear RSK2 and Erk1/2 were detected by anti-RSK2 or Erk1/2 immunofluorescent analysis. As shown in Figure 3C, RSK2 immunofluorescent staining was detected in both cytoplasmic and nuclear compartments in control M-RON cells. Upon MSP stimulation, increased nuclear fluorescent intensity was observed, indicating nuclear accumulation of RSK2 and Erk1/2. We noticed that RSK2 nuclear staining appeared as a pattern of condensed granules. Cellular distribution of Erk1/2 in control cells was similar to that of RSK2. MSP induced Erk1/2 nuclear translocation with increased nuclear fluorescent intensity. The patterns of Erk1/2 nuclear staining were in a relatively diffused manner. Consistent with these observations, RSK 2 nuclear accumulation also was observed in cells stimulated with MSP plus TGF-β1 with granule-like staining pattern. Again, Erk1/2 accumulated in nucleus with combined stimulation but distributed in a more diffused pattern. These results, together with those in Figure 3A and 3B, demonstrated that distribution and phosphorylation between RSK2 and Erk1/2 upon MSP stimulation exist.

### Preventive effect of RSK2 inhibitor SL0101 on MSP or MSP plus TGF-β1-induced EMT

**Figure 4:**
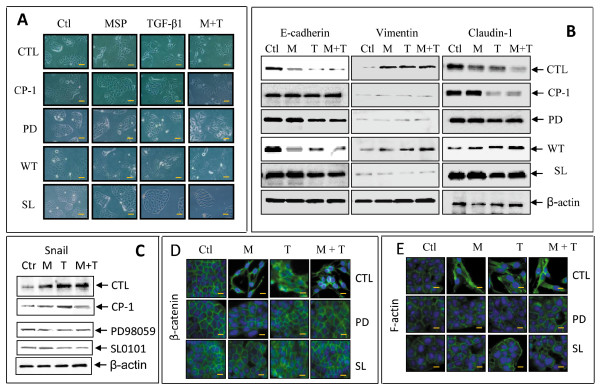
**Preventive Effect of RSK inhibitor SL0101 on MSP and MSP plus TGF-β1-induced EMT**: M-RON cells (1 × 10^5 ^cells/well in a 24-well plate) were incubated overnight and then stimulated with MSP (2 nM), TGF-β1 (5 ng/ml), or both at 37°C for 24 h. Small chemical inhibitors specific to RON (CP-1, 100 nM), Erk1/2 (PD98059, 100 μM), PI-3kinase (wortmannin, 50 μM), and RSK (SL0101, 50 μM) were added simultaneously. **A**) Cell morphological changes were observed and photographed using Olympus BK71 microscope equipped with CCD camera. Scale bars represent 20 μm. **B**) Cellular proteins (50 μg per sample) were also prepared for Western blot analysis. Expression of E-cadherin, claudin-1, and vimentin was determined by using specific antibodies. Membranes were also reprobed with antibodies to actin as the loading control. **C**) Transcription repressor Snail in nuclear proteins was detected by Western blot analysis using anti-Snail antibody. Preparation of nuclear factions was performed as previously described [45]. **D**) and **E**) β-catenin redistribution and F-actin reorganization was determined by immunofluorescent analysis [35] using antibodies specific to β-catenin and F-actin, respectively. Data shown here are from one of three experiments with similar results. Scale bars represent 10 μm.

To determine if RSK2 is indeed an effector molecule, we studied the effect of SL0101 on MSP-induced EMT. We also used TGF-β1 to induce EMT for evaluation. Results in Figure 4A showed that MSP induced spindle-like morphological changes in M-RON cells. As expected, this effect was prevented by CP-1 and PD98059, but not by PI-3 kinase inhibitor wortmannin. Consistent with results shown in Table 1, SL0101 significantly prevented MSP-induced spindle-like morphology. SL0101 also prevented TGF-β1-induced cell shape changes, but its effect was not complete. Moreover, the synergistic effect of MSP and TGF-β1 in cell morphology was affected by SL0101 (Figure 4A). In all these cases, altered cell morphology was significantly restored to original epithelial appearance.

Experiments were then conducted to determine if SL0101 regulates E-cadherin, claudin-1, and vimentin expression (Figure 4B). CP-1, PD98059, and wortmannin were included as controls. SL0101 completely prevented MSP-induced reduction of E-cadherin. Sl0101 also prevented increased vimentin expression. These observations concurred with results from cells treated with CP-1 and PD98059, but not with wortmannin, Additionally, SL0101 treatment restored claudin-1 expression, a protein essential for epithelial tight junction formation [40]. Preventive effect of SL0101 also was seen in M-RON cells stimulated with TGF-β1 and MSP plus TGF-β1. In both cases, expression of E-cadherin and claudin-1 was restored and induction of vimentin was blocked.

Activation of transcription repressor Snail is known to suppress E-cadherin expression leading to EMT [16,41]. Analysis of nuclear proteins from MSP-treated M-RON cells by Western blotting revealed that inhibition of RSK2 by SL0101 had a negative effect on RON-mediated Snail expression (Figure 4C). SL0101 prevented MSP-induced Snail expression in M-RON cells. Reduced Snail expression was also seen in MSP-stimulated cells treated with CP-1 and PD98059. Again, the action of SL0101 was not limited to MSP, as SL0101 also prevented TGF-β1-induced Snail expression. We want to emphasize that Snail expression induced by TGF-β1 was sensitive to PD98059 but not to CP-1 (Figure 4C).

We further studied the effect of SL0101 on MSP and TGF-β1-induced redistribution of β-catenin and F-actin. Both proteins play a role in RON-mediated EMT [30,35]. Results in Figure 4D showed the redistribution of β-catenin from cell membrane to cytoplasmic compartment upon MSP and TGF-β1 stimulation. SL0101 prevented MSP and TGF-β1-induced β-catenin redistribution and cytoplasm-associated β-catenin disappeared after addition of SL0101. A similar effect also was observed in cells treated with PD98059. In both cases, β-catenin was redistributed to cell membrane along with typical epithelial morphology. The effect of SL0101 on F-actin distribution was very similar to those of β-catenin after treatment with MSP, TGF-β1, and both (Figure 4E). F-actin was mainly associated with cell membrane with a certain amount of cytoplasmic distribution. MSP and TGF-β1 caused increased accumulation of F-actin in cytoplasm. This effect was prevented by SL0101, which restored F-actin distribution to its original membrane-associated appearance. This effect was also accompanied by the reappearance of epithelial morphology.

**Figure 5:**
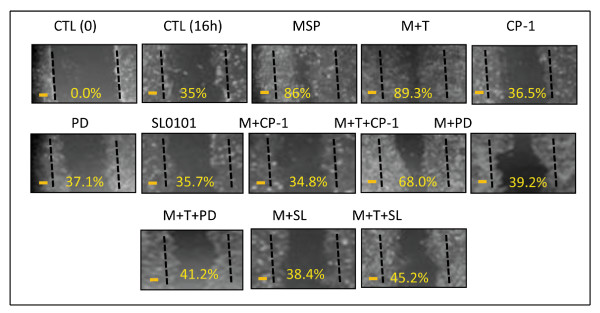
**Effect of SL0101 on MSP or MSP plus TGF-β1-induced cell migration**. The monolayer of M-RON cells in DMEM with 1% FBS) were wounded with a plastic tip and then stimulated with MSP (2 nM), TGF-β1 (5 ng/ml), or both in the presence or absence of CP-1 (300 μg/ml), PD98059 (100 μM), and SL0101 (50 μM). After incubation for 16 h, cells that migrated into the open spaces were observed under microscope and photographed. Wounded areas that covered by migrated cells were calculated as previously described [35]. Data shown here are from one of two experiments with similar results. Scale bars represent 50 μm.

We performed the wound-healing assay to determine if SL0101 can prevent MSP-induced migration of M-RON cells. Increased migration is a function associated with EMT. Results in Figure 5 showed that M-RON cells had spontaneous migration (35.0%) and MSP stimulation further enhanced cell motility (86.0%). Treatment of cells with SL0101 alone had no effect on cell migration; however, SL0101 significantly prevented MSP or MSP-plus TGF-β1-induced cell migration. The percentages of cell migration induced by MSP and MSP plus TGF-β1 (86.0% and 89.3%, respectively) were dramatically reduced after SL0101 treatment (38.4% and 45.2%, respectively). We observed inhibition levels that were comparable to those treated with CP-1 and PD98059. Thus, results in Figure 4 and 5 demonstrated that SL0101 inhibition of RSK prevented MSP and TGF-β1-induced spindle-like morphology accompanied with redistribution of β-catenin and F-actin. E-cadherin and claudin-1 expression reappeared and vimentin expression was blocked. These activities were associated with the inhibition of transcription repressor Snail expression. Moreover, SL0101 significantly impairs MSP and TGF-β1 induced cell migration, which is a function associated with EMT.

### Effect of increased RSK expression in MSP-induced EMT-like activity in cancer cells

**Figure 6:**
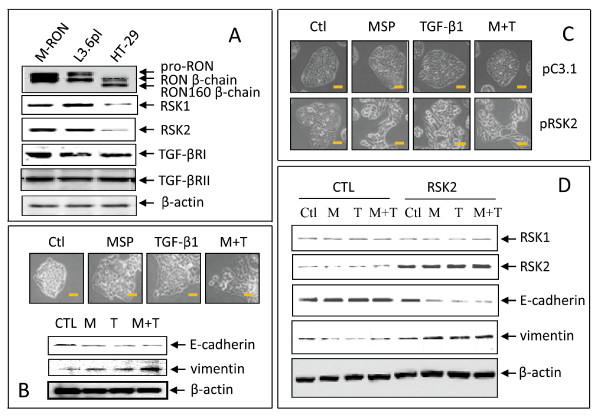
**Requirement of RSK2 expression in MSP and TGF-β1 induced EMT-like activity in cancer cells**. **A**) Expression of RON, RSK1, RSK2, and TGF-β receptors in human cancer L3.6pl and HT-29 cells. Cell lysates (50 μg/sample) were subjected to Western blot analysis using antibodies specific to individual proteins. **B**) MSP induces EMT-like activities in pancreatic cancer L3.6pl cells. L3.6pl cells (0.5 × 10^6 ^cells per dish) were cultured overnight and then stimulated at 37°C with MSP (2 nM), TGF-β1 (5 ng/ml), or both for 24 h. Cell morphological changes were observed by Olympus microscope and photographed with CCD camera. E-cadherin and vimentin expression was determined by Western blot analysis using cell lysates as described previously [35]. Actin was used as the loading control. Scale bars represent 20 μm. **C**) Forced RSK2 expression facilitates MSP and TGF-1-induced EMT-like activity in HT-29 cells. Cells (2 × 10^6 ^cells per dish) were transiently transfected with 3 μg of pRKS2 plasmid or control vector pcDNA3.1 for 48 h and then stimulated with MSP and TGF-β1 as described above. Morphological changes and expression of individual proteins were determined as described in A. Scale bars represent 20 μM. **D**) RSK2 expression diminishes E-cadherin expression and increases vimentin expression. HT-29 cells were transiently transfected with pRSK2 plasmid for 48 h followed by stimulation with MSP, TGF-β1 or both for 24 h. Cell lysates were subjected to Western blot analysis using antibodies specific to E-cadherin or vimentin. β-actin was used as the loading control. Data shown here are from one of two experiments with similar results.

To study the effect of RSK2 on MSP-induced EMT in more detail, two human cancer cell lines L3.6pl and HT-29 were selected based on their differences in RSK1 and RSK2 levels and similarities in RON and TGF-β receptor expression (Figure 6A). Pancreatic cancer L3.6pL cells expressed regular levels of RSK1 and RSK2. MSP and TGF-β1 stimulation caused elongated cell morphology, reduced E-cadherin expression, and increased vimentin expression (Figure 6B). Combined MSP and TGF-β1 treatment further enhanced the modulating effect on E-cadherin and vimentin expression. These results indicated that L3.6pl cells show EMT-like phenotypic changes after MSP and TGF-β1 stimulation and a synergistic activity between RON and TGF-βRI/II signaling in induction of EMT-like phenotype.

HT-29 cells expressed extremely low levels of RSK1 and RSK2 (Figure 6A). Treatment of cells with MSP, TGF-β1 or both caused barely any morphological changes (data not shown). Western blot analysis also failed to observe any changes in E-cadherin and vimentin expression in MSP plus TGF-β1-stimulated HT-29 cells (data not shown). However, RSK2 overexpression by pRSK2 plasmid transfection resulted in cell morphological changes after MSP stimulation (Figure 6C). We observed similar changes when transfected HT-29 cells were stimulated with TGF-β1 or MSP plus TGF-β1. Analysis of E-cadherin and vimentin expression in pRSK2-transfected HT-29 cells confirmed that MSP and TGF-β1 stimulation caused E-cadherin reduction and vimentin induction (Figure 6D). These results suggested that increasing RSK2 expression renders HT-29 cells responsive to MSP and TGF-β1-induced EMT-like activities.

### Effect of RSK specific siRNA on MSP-induced cell migration

**Figure 7:**
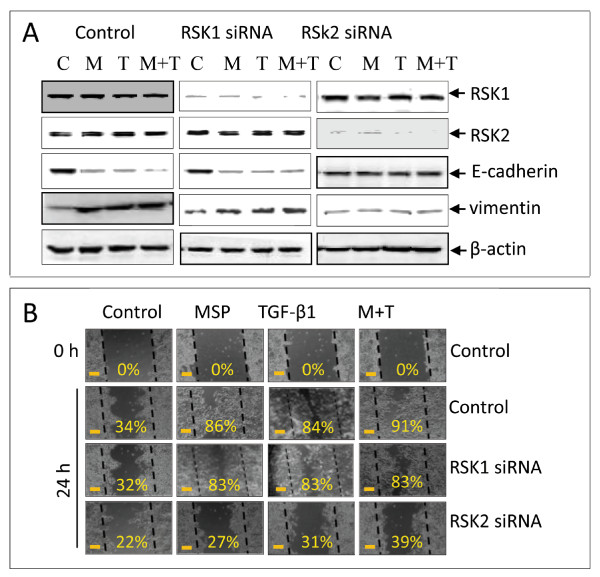
**Effect of siRNA-mediated RSK2 knockdown on MSP and TGF-β1-induced EMT-like activities and cell migration by L3.6pl cells: A**) Knockdown of RSK2 expression by specific siRNA restored E-cadherin expression and prevented vimentin induction. L3.6pl cells (2 × 10^6 ^cells per dish) were cultured overnight and transiently transfected with 3 μg/dish of pcDNA3.1, pRSK1, or pRSK2 by using lipofectamine (Invitrogen). Transfected cells were cultured for 72 h and then lysed with lysis buffer. Cellular proteins (50 μg per sample) were subjected to Western blot analysis as described previously using antibodies specific to RSK1, RSK2, E-cadherin, and vimentin, respectively. The membranes were also reprobed with rabbit IgG to actin as the loading control. **B**) L3.6pl cells were transiently transfected with pcRSK1 or pcRSK2 for 48 h followed by stimulation with MSP, TGF-β1, or both as described above. The wound healing assay was performed after a 24 h-incubation to determine the levels of cell migration. The percentage of wounded area covered by migrated cells was determined as previously described [45]. Data shown here are from one of three experiments with similar results. Scale bars represent 50 μm.

To further confirm the role of RSK2, we transiently transfected L3.6pl cells with specific siRNA to silence RSK1 or RSK2 mRNA expression. Results in Figure 7A showed that siRNA specific to RSK1 effectively silenced RSK1 expression but had no effect on RSK2 expression. RSK2 specific siRNA only silenced RSK2 expression but had no effect on RSK1 expression. These results confirmed specificities of siRNA used to silence RSK1 and RSK2, respectively. Analysis of MSP and TGF-β1-regulated epithelial and mesenchymal proteins revealed that silencing RSK1 expression did not prevent MSP and TGF-β1-induced reduction of E-cadherin and induction of vimentin. In contrast, knockdown of RSK2 expression restored E-cadherin expression and prevented vimentin induction. We also observed these effects in cells treated with TGF-β1 and MSP plus TGf-β1, indicating that RSK2 was required for MSP and TGF-β1-induced EMT-like biochemical changes.

We further studied the effect of siRNA mediated RSK2 knockdown on cell migration by the wound healing assay (Figure 7B). L3.6pl cells showed spontaneous migration, which was further enhanced by MSP stimulation. The amount of open space covered by migrated cells increased from 34% up to 86%. Knockdown of RSK1 had little effect on spontaneous cell migration, but silencing RSK2 expression showed a moderate effect on spontaneous cell migration. In MSP-induced cell migration, silencing RSK1 expression did not impair MSP-induced cell migration, as more than 80% of the open space was still covered by migrated cells. In contrast, MSP-induced cell migration was significantly impaired in RSK2 siRNA treated cells. In this case, only 27% of the open space was covered by migrated cells, which was similar to spontaneous migration. TGF-β1-induced cell migration was not affected by knockdown of RSK1. The inhibitory effect was only observed in cells treated with specific RSK2 siRNA. Moreover, we observed that silencing RSK2 expression also impairs cell migration synergized by combined MSP and TGF-β1 stimulation. Thus, silencing RSK2 but not RSK1 by specific siRNA decreases MSP-induced cell migration in L3.6pl cancer cells.

## Discussion

The purpose of this study is to identify the major signaling molecule(s) that controls MSP-induced EMT in epithelial cells. Altered RON expression and activation contribute to malignant progression of various epithelial cancers [30,42]. RON is overexpressed in various types of primary cancer samples including those from colon, breast, and pancreas [42]. Aberrant RON activation also causes increased tumor cell proliferation, matrix invasion, and drug resistance [42]. Currently, the role of MSP and RON in regulating EMT under physiological conditions is largely unknown. In contrast, MSP-induced RON activation or RON overexpression have been shown to induce EMT in various cancer cells including colon, breast, and pancreas [30,31,43-45]. The changes to mesenchymal phenotype in RON-activated tumor cells have been considered as a molecular basis for increased tumor malignancy including cell migration, matrix invasion, and distance metastasis [42]. Several upstream signaling proteins such as Erk1/2 have been implicated in MSP-induced EMT [30,31]; however, the major effector molecule(s) that transduces RON signals leading to EMT is still unknown. Intracellular proteins such as β-catenin and NF-κB have been identified as effector molecules in MSP-induced EMT [45-47]. Nevertheless, their significance is often limited to particular cell models. Thus, identification of the major signaling molecule(s) is important not only for an understanding of the cellular mechanisms of EMT, but also for the development of potential therapies that target cancer cell migration and invasion.

Results from this study indicate that RSK2 is a major determinant bridging RON signaling to EMT. This conclusion is supported by the following evidence. First, inhibition of RSK, as indicated in the cell-shape based screen by using specific RSK inhibitor SL0101, completely prevented MSP-induced spindle-like morphology. Inhibitors that target other proteins such as NF-κB, Stat3, and hedgehog, except CP-1 and PD98059, only showed moderate effect. This indicates that RSK activation is essential in MSP-induced spindle-like morphology. Second, MSP-induced RON activation dissociated RSK2 from Erk1/2, and caused RSK2 phosphorylation and subsequent nuclear translocation. These data suggest that MSP is a strong RSK activation inducer, which is mediated by RON transduced signals. Third, RSK2 phosphorylation relied on the RON-Erk1/2 pathways. Inhibition of RON or Erk1/2 by their corresponding small chemical inhibitors prevented MSP-induced RSK2 phosphorylation. These data also established that RSK is a downstream molecule in the MSP-RON-Erk1/2 axis. Fourth, inhibition of RSK2 by SL0101 blocked MSP-induced spindle-like changes, which is evident by the redistribution of β-catenin to the membrane and reorganization of f-actin to original epithelial morphology. Moreover, in SL0101 treated cells, epithelial morphology was completely restored with re-expression of E-cadherin and claudin-1, reduction of vimentin expression, and minimized transcription repressor Snail expression. Fifth, SL0101 prevention of RSK2 activation decreased MSP and TGF-β1-induced cell migration. As shown in the wound healing assay, RON-mediated cell migration was dramatically reduced upon inhibition of RSK2 by SL0101. Finally, RSK2 overexpression led to EMT-like phenotypes in colon HT-29 cancer cells that express extremely low levels of RSK2. Moreover, specific siRNA-mediated RSK2 knockdown prevented MSP and TGF-β1-induced EMT-like activity in pancreatic cancer L3.6pl cells. Considering these factors, we concluded that SRK2 is the major effector molecule in RON-mediated EMT.

In reviewing cellular mechanisms underlying EMT in different types of epithelial and cancerous cells, it is apparent that various proteins belonging to multiple signaling pathways are involved in regulating EMT [4,5]. The identified proteins include Erk1/2, PI-3 kinase, AKT, p38, β-catenin, NF-κB, Stat3, Smad, and others [11-20]. The typical example is the Erk1/2-mediated signaling event that leads to EMT [17,22]. Specifically, Erk2 but not Erk1 has been found to be critical in EMT induction, which is mediated by DEF motif-dependent signaling events [17]. Currently, the signaling proteins participated in EMT represent at least seven different signaling pathways. The involvement of such diverse signaling proteins suggests the possible existence of a central signaling molecule that acts as a switch for initiation of EMT in epithelial cells. In supporting this notion, recent studies has shown that RSK acts as a principal effector molecule in coordinating cellular EMT program in epithelial cells [22]. Genome-wide RNAi screen also has discovered that multiple proteins in a broad range of pathways depend on RSK for induction of cellular migration program [23]. We observed that RSK2 activation is critical in controlling EMT in MDCK and cancer cells mediated by MSP. Moreover, RSK2 is also required for TGF-β1-induced EMT. The involvement of RSK2 in two different signaling pathways suggests that RSK2 acts as a potential central molecule in regulating EMT and cell migration. In other words, RSK2 activation acts as the convergent point for both RON-Erk1/2 and TGF-β receptor I/II-Smad pathways leading to complete EMT.

The importance of RSK2 in RON signaling also establishes a critical link to other signaling molecules observed in MSP-induced EMT and cell migration. Activation of Erk1/2 is required for MSP-induced EMT [30,31]. As a downstream molecule of the Erk1/2 pathway, RSK2 transduces MSP-induced and Erk1/2 mediated signal for EMT as demonstrated in this study. In breast cancer cells, NF-κB activation is implicated in RON-mediated cellular motility [47]. RSK is known to activate NF-κB by phosphorylating NF-κB inhibitor IκBα and inducing its degradation [48]. This finding suggests that the observed NF-κB activity in MSP-stimulated breast cancer cells could be channeled through RON-activated RSK2. In colon cancer cells stimulated by MSP, increased β-catenin accumulation contributes to spindle-like morphologies with increased migration [35]. RSK2 activation is known to increase steady-state of β-catenin through phosphorylation and inhibition of a β-catenin regulator GSK-3β [49]. These activities imply that the RON-mediated inhibition of GSK-3β [35] could be caused by MSP-induced RSK2 activation. The role of MSP-activated AKT activity in cell migration is another example [34]. Currently, evidence of direct RSK activation by AKT is not available. In contrast, studies have indicated that RSK is a mediator of growth factor-induced activation of PI-3 kinase and AKT in epithelial cells [50]. Thus, it is likely that MSP-induced AKT activation is mediated by RSK. Such activation facilitates AKT in regulating MSP-induced cell migration. Considering all these facts, we reasoned that RSK is centered in MSP-induced and RON-mediated EMT with increased cell migration.

Studies sing pancreatic L3.6pl and colon HT-29 cells provide additional evidence showing the importance of RSK2 in MSP-induced EMT-like activity. First, we confirmed results derived from the MDCK cell model and demonstrated that RSK2 but not RSK1 is selectively involved in regulating RON-mediated EMT and associated cell migration. In the L3.6pl cell model, only RSK2 specific siRNA prevented MSP-induced EMT and cell migration. Second, we demonstrated that MSP-induced EMT-like phenotype is dependent on RSK2 expression and activation. In L3.6pl cells that express regular levels of RSK1 and RSK2, MSP induces EMT-like phenotypes featured by elongated cell morphology, reduced E-cadherin expression, and increased vimentin expression (Figure 6). In contrast, these activities were not observed in HT-29 cells that express minimal levels of RSK1 and RSK2. HT-29 cells express both RON and oncogenic variant RON160 and both regulate HT-29 cell growth [51]. However, MSP fails to induce EMT and migration in HT-29 cells, which provides indirect evidence indicating the role of RSK2 in MSP-induced EMT and cell migration. Rescue experiments by pRSK2 cDNA transfection confirmed this theory. As shown in Figure 6C, RSK2-transfected HT-29 cells underwent spindle-like morphological changes with diminished E-cadherin and increased vimentin expression. Additional evidence supporting this notion comes from studies using RSK2-specific siRNA. Knockdown of RSK2 expression significantly inhibited MSP-induced L3.6pl cell migration (Figure 7), which reaffirms the importance of RSK2 in MSP-induced EMT. The final observation is that the effect of RSK2 on EMT is not limited to MSP. TGF-β1-induced EMT and cell migration also were affected by inhibition of RSK2. HT-29 cells with minimal RSK2 expression did not respond to TGF-β1. Spindle-like morphology was only seen when RSK2 is overexpressed. Western blot analysis of E-cadherin and vimentin expression in RSK2 deficient and transfected HT-29 cells confirmed that this is the case. RSK2 siRNA based analysis of cell migration further demonstrated that knockdown of RSK2 expression significantly impairs TGF-β1-induced L3.6pl cell migration.

## Conflict of interests

The authors declare that they have no competing interests.

## Authors' contributions

QM performed the majority of biochemical analysis and biological experiments. SG and SSP did cellular immunofluorescent studies. HPY worked on anti-RON antibody production and characterization, RWZ, YQZ, and MHW participated in the design of the study and drafted the manuscript. All authors have read and approved the final manuscript.
